# Association between Elevated Hemoglobin A1c Levels and the Outcomes of Patients with Small-Artery Occlusion: A Hospital-Based Study

**DOI:** 10.1371/journal.pone.0160223

**Published:** 2016-08-03

**Authors:** Yuan Gao, Lihong Jiang, Hui Wang, Changshen Yu, Wanjun Wang, Shoufeng Liu, Chunlin Gao, Xiaoguang Tong, Jinhuan Wang, Yi Jin, Jialing Wu

**Affiliations:** 1 Department of Neurology, Tianjin Huanhu Hospital, Tianjin Key Laboratory of Cerebrovascular and Neurodegenerative Diseases, Tianjin, China; 2 Department of Neurosurgery, Tianjin Huanhu Hospital, Tianjin Key Laboratory of Cerebrovascular and Neurodegenerative Diseases, Tianjin, China; 3 Department of Nursing, Tianjin Huanhu Hospital, Tianjin Key Laboratory of Cerebrovascular and Neurodegenerative Diseases, Tianjin, China; Baylor College of Medicine, UNITED STATES

## Abstract

**Introduction:**

Abnormal glucose metabolism is an independent risk factor for poor outcome following acute ischemic stroke. However, the relationship between initial hemoglobin A1c level and functional outcome (defined by modified Rankin Scale scores) following small-artery occlusion, a subtype of ischemic stroke, is unknown. The aim of the present study was to evaluate this association among patients diagnosed with small-artery occlusion.

**Materials and Methods:**

Data on 793 patients diagnosed with small-artery occlusion from October 25, 2012 to June 30, 2015 were collected from the stroke registry of the Department of Neurorehabilitation of HuanHu Hospital. Hemoglobin A1c values at admission were classified into three groups according to tertiles (<5.9,5.9to<6.7, and**≥**6.7). We used receiver operating characteristics curves to investigate the predictive value of hemoglobin A1c and examined the relationship between hemoglobin A1c levels at admission and modified Rankin Scale scores using univariate and multivariate analyses.

**Results:**

The area under the curve was 0.570 (95%CI, 0.509–0.631; *P* = 0.023). Patients in the highest HbA1c stratification (**≥**6.7) had a significantly higher risk of an unfavorable outcome than patients in the lowest stratification (<5.9; adjusted odds ratio, 2.099; 95%CI, 1.160–3.798; *P* = 0.014). However, a significant association was not seen in the middle stratification (5.9 to <6.7; *P* = 0.115).

**Conclusions:**

Elevated hemoglobin A1c level on admission was adversely associated with functional outcomes 3 months after stroke onset among patients presenting with small-artery occlusion.

## Introduction

Ischemic stroke is a heterogeneous disease, and small-artery occlusion (SAO) is a subtype of ischemic stroke classified by the Trial of ORG10172 in Acute Stroke Treatment (TOAST). The age-standardized incidence of SAO for the European population is 25.8%[1]. In China, SAO accounts for 31.3% of ischemic stroke cases[2]. SAO is the leading cause of functional loss in the elderly[3]. Patients diagnosed with SAO may live much longer lives than patients diagnosed with other ischemic stroke subtypes because SAO is associated with the lowest stroke severity and mortality rate among stroke subtypes[4].Therefore, functional independency, evaluated using the modified Rankin scale (mRS), is more meaningful for survivors than other outcome measures. Both diabetes mellitus and pre-diabetes mellitus are independent risk factors for poor outcomes following acute ischemic stroke. A large number of studies utilized mortality rate as an outcome measure and demonstrated that hemoglobin A1c (HbA1c) levels may affect ischemic stroke patients’ outcomes[5,6,7,8,9,10]. The association between HbA1c levels and functional outcomes has not been established, especially among SAO patients. Evaluating this relationship can help us further understand the pathophysiological role of pre-stroke glycemic control status among SAO patients and provide reference for clinical practice.

HbA1c levels, which may not be affected by confounding variables such as the presence of stress hyperglycemia caused by stroke, reinstitution of oral feeding, or intravenous administration of glucose-containing fluids[11], reflects long-term glycemic control and are a more accurate and stable measure of glycemic control than random blood glucose testing[12]. Therefore, most patients are advised to measure HbA1c concentration in clinical practice, and it is also a widely accepted parameter for describing pre-stroke glycemic control status[13,14].

The aim of the present study was to investigate the association between HbA1c levels on admission and mRS scores after SAO.

## Materials and Methods

### Ethics

The study was approved by the ethics committee of Tianjin Huanhu Hospital, and written informed consent was obtained from each subject. All procedures involving human participants were in accordance with the ethical standards of the ethics committee of Tianjin Huanhu Hospital and with the 1964 Helsinki Declaration and its later amendments or comparable ethical standards.

### Patient selection

We utilized a hospital-based registry that included consecutive patients with stroke who visited the Department of Neurorehabilitation of HuanHu Hospital. Detailed baseline data were collected and abstracted using electronic-based record forms during patients’ hospitalization. Patients or their authorized proxies were followed for 3 months after stroke onset through either telephone or in-person interviews. Follow-up results were immediately incorporated into the stroke database.

**Fig 1:**
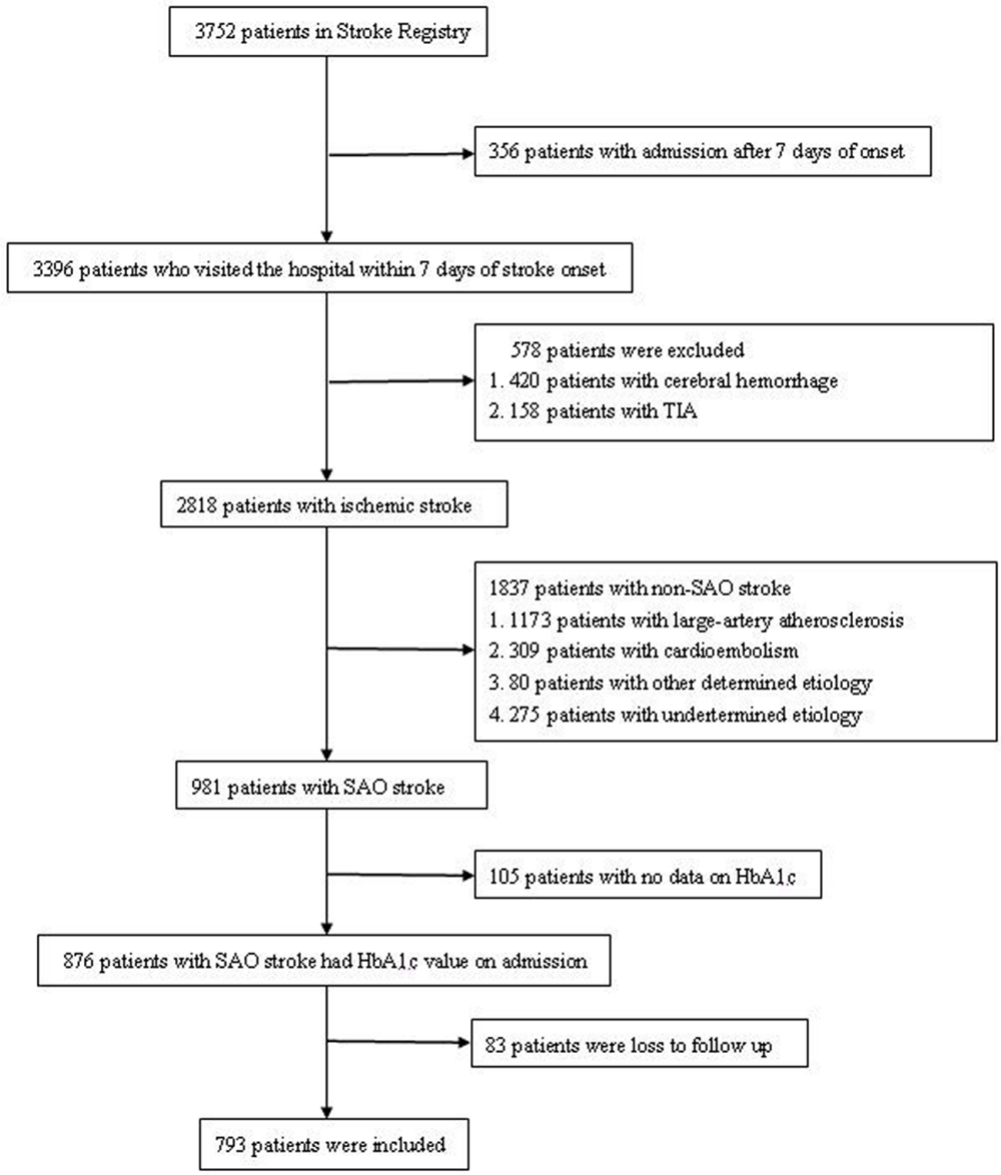
Flow chart of patient selection. SAO: small-artery occlusion; HbA1c: hemoglobin A1c.

We retrospectively reviewed and extracted data from the stroke registry between October 25, 2012 and June 30, 2015. The selection procedure in the present study was as follows. Among a total of 3752 patients,356 patients who visited the hospital after 7 days were excluded. Among the remaining patients(n = 3396), those with cerebral hemorrhage(n = 420) or transient ischemic attack(n = 158) were excluded. A total of 2818 patients were diagnosed with ischemic stroke according to World Health Organization criteria[15]; diagnoses were confirmed by brain computed tomography or magnetic resonance imaging. Ischemic stroke was classified into five subtypes according to the TOAST classification criteria[16].Among the 2818 participating patients,981 were diagnosed with SAO. After excluding patients with no HbA1c data on admission(n = 105) and those who were lost to follow-up(n = 83), 793 patients with SAO were analyzed in this study (Fig 1).

SAO was defined as an infarction presenting with the following features[16]. Patients were required to have one of the traditional clinical lacunar syndromes with no evidence of cerebral cortical dysfunction. The infarction was required to have been located in the subcortex or brain stem and be <1.5 cm in diameter on computed tomography/magnetic resonance imaging. A history of diabetes mellitus or hypertension supported the clinical diagnosis. Patients were required to have no potential cardiac sources for embolism and stenosis no greater than 50% in an ipsilateral artery. The diagnosis of SAO was performed independently by two experienced neurologists.

### Demographic and clinical assessment

All patients underwent routine diagnostic testing, and their baseline information and stroke risk factors were collected within 24h of admission. Baseline information, including age, sex, medical history, and detailed demographics data, were collected. Blood biochemical variables were measured following a fast of at least 8h on the first day after admission. We routinely test HbA1c on admission, as diabetes is an important risk factor for stroke and this can provide information to help us further screen and diagnose patients with diabetes. All variables were analyzed in a certified central laboratory.

HbA1c values were classified into three groups according to tertiles (<5.9,5.9 to <6.7,and **≥**6.7). In the pre-analysis, we also classified HbA1c values into four groups according to quartiles(<5.8,5.8to<6.2,6.2to<7.1, and **≥**7.1), because tertile classification and quartile classification are the most common statistical layering methods. Diabetes was defined as a previous diagnosis of diabetes and/or the use of hypoglycemic agents, with an HbA1c level ≥6.5%[17]. Hypertension was defined as either receiving antihypertensive treatment or a blood pressurelevel≥140/90 mmHg on repeated measurements[18]. Dyslipidemia was defined as either receiving treatment with cholesterol-reducing agents, a triglyceride level>2.26mmol/L(200mg/dL), or a total cholesterol level >6.21mmol/L (240mg/dL) [19]. Patients who smoked tobacco products almost every day for>1year were defined as current smokers. Patients who consumed alcohol at least one time per week for>1year were defined as current drinkers. Body mass index was measured at the time of admission, and obesity was defined as a body mass index≥30 kg/m^2^. The National Institutes of Health Stroke Scale (NIHSS) was used to assess severity of stroke[20].

During hospitalization, treatment and target levels followed national and international guidelines[17,21,22]; treatments included anti-thrombotic agents(aspirinor clopidogrel), anti-hypertension agents (calcium channel blockers, angiotensin-converting enzyme inhibitors, diuretics, angiotensin receptor blockers, or beta receptor blockers), glucose-lowering agents (biguanides, sulfonylureas, glinides, alpha glucosidase inhibitors, TZD, insulin and its analogues, GLP-1 agonists, DPP-4 inhibitors), and lipid-lowering agents (statins). The details of medications used during hospitalization were recorded in electronic-based records.

For all patients diagnosed with diabetes, plasma glucose levels were maintained within the normal range with insulin or an oral hypoglycemic agent, according to Standards of Medical Care in Diabetes[17]. Fasting glucose was measured individually each day to show the effect of monitoring for hypoglycemia, and the results were also logged in patients’ electronic-based medical records. The average of three fasting glucose measurements taken on three consecutive days before discharge was used as another criterion to assess glucose metabolism.

### Study outcome

To evaluate functional outcome, functional impairment was graded using the mRS. A favorable outcome was defined as an mRS score of 0–2 at 3 months after stroke onset, indicating that a patient had no or only slight disability while remaining functionally independent. A poor outcome (lost functional independence) was defined as an mRS score of 3–5[23]. We checked the records of patients in the community registry, and there were no death records during the follow-up period.

### Statistical analysis

Continuous variables are presented as means±standard deviations. The significance of intergroup differences was assessed using the *t*-test and one-way analysis of variance. Categorical variables are presented as counts and percentages. The chi-square test was used to analyze differences between groups.We used receiver operating characteristics(ROC) curves to investigate the predictive value of HbA1c, and we calculated the areas under the curve (AUCs).Variables such as age, sex, hypertension, dyslipidemia, alcohol consumption, smoking, obesity, and NIHSS score were considered confounders. In the univariate analysis, we compared the difference between the favorable outcome group and poor outcome group. Variables of confounders that were identified as significant in the univariate analyses(*P*<0.05) were entered into logistic regression analyses to determine the association between HbA1c levels and mRS scores.

The software package SPSS 17.0 was used to perform statistical analyses. Statistical significance was defined as a (two-sided) *P* value<0.05.

## Results

### ROC curve of HbA1c and poor outcome

**Fig 2:**
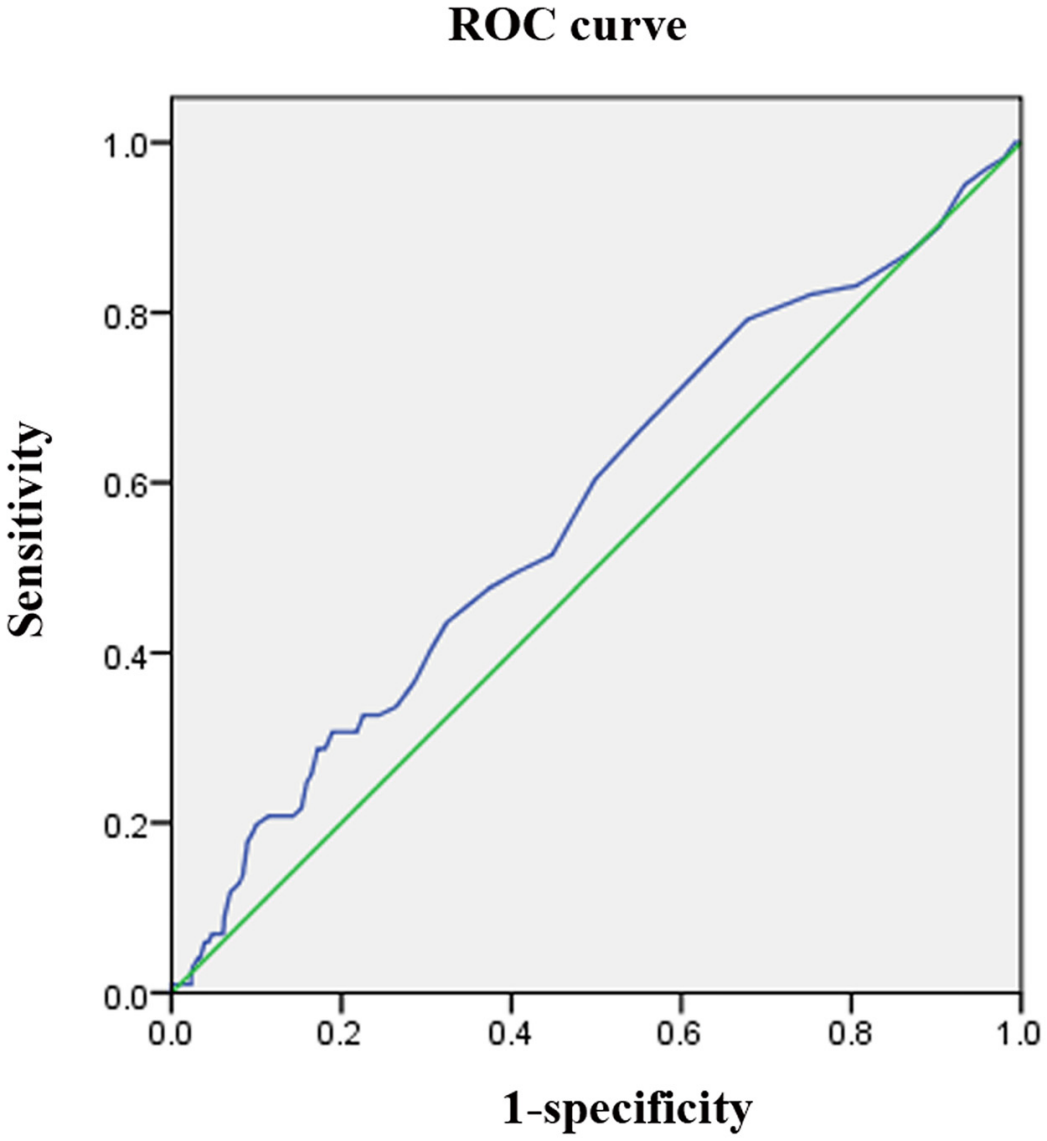
ROC curve of HbA1c and poor outcome. ROC: receiver operating characteristics.

The ROC curve(Fig 2) demonstrated that HbA1c has the potential to predict outcome 3 months after stroke onset. The AUC was 0.570 (95% CI, 0.509–0.631; *P* = 0.023).

### Baseline demographics and clinical characteristics

Baseline characteristics and clinical characteristics according to the HbA1c tertiles are presented in Table 1. Seven hundred ninety-three patients(551 men[69.5%]; mean age, 61.7±11.1years; range,30–93 years), including 692 patients (87.3%) with favorable outcome and 101 patients (12.7%) with poor outcome, were included in the study; 105 SAO patients, including 80 patients (81.0%) with favorable outcome and 25 patients (19%) with poor outcome, were excluded because their HbA1c levels were not tested on admission. There was no statistical association in the distribution between the two parts (*P* = 0.075).

**Table 1:** Baseline demographics and clinical characteristics according to HbA1c tertiles.

	Tertiles of HbA1c, mmol/L		
	< 5.9 (*n* = 244)	5.9 to <6.7 (*n* = 281)	≥ 6.7 (*n* = 268)
**Age, years (median values)** *	59.99 ± 11.75	62.28 ± 11.19	62.56 ± 10.34
Male sex, n(%)*	190 (77.9)	195 (69.4)	166 (61.9)
**Risk factors**			
Hypertension, n(%)*	171 (70.1)	231 (82.2)	197 (73.5)
Dyslipidemia, n(%)*	62 (25.4)	65 (23.1)	88 (32.8)
Smokers, n(%)*	123 (50.4)	105 (37.4)	87 (32.5)
Alcohol drinkers, n(%)*	68 (27.9)	41 (14.6)	40 (14.9)
Obesity, n(%)*	13 (5.3)	34 (12.1)	42 (15.7)
**Laboratory findings (median values)**			
**TC, mmol/L**	4.99 ± 2.81	4.81 ± 1.00	5.09 ± 1.04
TG, mmol/L*	1.60 ± 1.03	1.63 ± 0.88	1.95 ± 1.18
HDL-C, mmol/L*	1.09 ± 0.30	1.06 ± 0.26	1.02 ± 0.25
**LDL-C, mmol/L**	2.96 ± 0.77	2.90 ± 0.78	3.04 ± 0.82
HbA1c,mmol/L*	5.54 ± 0.24	6.17 ± 0.21	8.18 ± 1.39
Fasting glucose*	4.94 ± 0.77	5.49 ± 0.98	8.47 ± 2.70
**mRS on admission**			
**0–2**	177 (72.5)	188 (66.9)	183 (68.3)
**≥3**	67 (27.3)	93 (33.1)	85 (31.7)
**NIHSS, n(%)**			
**0–6**	192 (78.7)	229 (81.5)	215 (80.2)
**≥7**	52 (21.3)	52 (18.5)	53 (19.8)

* Indicates P< 0.05 when comparing between three groups. TC: total cholesterol; TG: triglyceride; HDL-C: high-density lipoprotein cholesterol; LDL-C: low-density lipoprotein cholesterol; HbA1c: hemoglobin A1c; mRS: modified Rankin Scale; NIHSS: National Institute of Health stroke scale

The mean HbA1c level of all patients was 6.7±1.4mmol/L (range,4.6–15.6mmol/L). NIHSS scores ranged from 1to14. Patients with HbA1c levels in the higher stratification were older than those in the lower stratification. Compared to women, men were less likely to have HbA1c levels in the higher stratification. In addition, patients with hypertension and dyslipidemia were unequally distributed among the HbA1c stratifications. Patients with HbA1c levels in the higher stratification were less likely to consume alcohol or smoke, while they were more likely to be obese. HbA1c levels in the higher stratification were associated with higher triglyceride levels, higher fasting glucose levels, and lower high-density lipoprotein cholesterol levels. There was not a statistically significant difference among the stratifications with regard to mRS and NIHSS scores at baseline.

### Association between HbA1c levels and functional outcomes

Table 2 compares the characteristics between the favorable outcome and unfavorable outcome groups. Among all patients with SAO, age, sex, HbA1c level (tertiles), smoking status, alcohol consumption, and NIHSS score were significantly different according to mRS score (*P*<0.001, *P =* 0.034, *P =* 0.008, *P =* 0.004, *P =* 0.014, and *P*<0.001, respectively).These variables were entered into logistic regression analyses. Table 3 shows the results of the logistic regression analyses of HbA1c levels and mRS scores. Patients in the highest HbA1c stratification had a significantly higher risk of an unfavorable outcome than patients in the lowest stratification, after adjusting for risk factors (i.e., age, sex, smoking status, alcohol consumption, and NIHSS score) identified as significant in the univariate analysis (adjusted odds ratio, 2.099; 95%CI, 1.160–3.798; *P* = 0.014). However, compared with the lowest HbA1c stratification, a significant association was not seen in the middle stratification (5.9 to <6.7; *P* = 0.115). The same statistical process was conducted in the pre-analysis; HbA1c levels(quartiles) were also significantly different between mRS score groups in the univariate analysis(*P* = 0.031). Using the quartile classification of HbA1cin logistic regression analyses, compared to the lowest stratification (<5.8), the highest stratification (**≥**7.1) was associated with a risk of poor outcome (adjusted odds ratio, 1.844; 95%CI, 1.000–3.403; *P* = 0.050), but a significant association was not seen with the middle two stratifications (5.8to< 6.2 and 6.2 to<7.1; *P =* 0.633, and *P* = 0.204, respectively).

**Table 2:** Comparison of the risk factors between groups classified by outcomes.

	mRS ≤ 2 (*n* = 692)	mRS ≥ 3 (*n* = 101)	*P* Value
**Age, years (median values)**	60.98 ± 10.84	66.38 ± 12.00	<0.001
**Male sex, n(%)**	490 (70.8)	61 (60.4)	0.034
**HbA1c level (tertiles)**			0.008
**< 5.9**	223 (32.2)	21 (20.8)	
**5.9 to < 6.7**	245 (35.4)	36 (35.6)	
**≥ 6.7**	224 (32.4)	44 (43.6)	
**HbA1c level (quartiles)**			
**< 5.8**	170(24.6)	18(17.8)	0.031
**5.8 to < 6.2**	177(25.6)	22(21.8)	
**6.2 to < 7.1**	176(25.4)	28(27.7)	
**≥ 7.1**	169(24.4)	33(32.7)	
**Hypertension, n(%)**	520 (75.1)	79 (78.2)	0.502
**Dyslipidemia, n(%)**	190 (27.5)	25 (24.8)	0.568
**Smokers, n(%)**	288 (41.6)	27 (26.7)	0.004
**Alcohol drinkers, n(%)**	139 (20.1)	10 (9.9)	0.014
**Obesity, n(%)**	72 (10.4)	17 (16.8)	0.056
**Fasting glucose**	6.19 ± 2.28	6.71 ± 2.28	0.056
**NIHSS, n(%)**			<0.001
**0–6**	584 (84.4)	52 (51.5)	
**≥7**	108 (15.6)	49 (48.5)	

mRS: modified Rankin scale; HbA1c: hemoglobin A1c; NIHSS: National Institute of Health stroke scale

**Table 3:** Multivariate logistical regression analyses of HbA1c levels and the outcome variables.

	B	OR (95% CI)	*P* Value
**Age**	0.348	1.417 (1.120–1.792)	0.004
**Male**	0.048	1.049 (0.837–1.316)	0.677
**HbA1c level (tertiles)**			
**< 5.9**			
**5.9 to< 6.7**	0.487	1.628 (0.888–2.984)	0.115
**≥ 6.7**	0.741	2.099 (1.160–3.798)	0.014
**Smokers**	-0.055	0.946 (0.717–1.248)	0.695
**Alcohol drinkers**	-0.088	0.915 (0.676–1.240)	0.567
**NIHSS**	0.610	1.840(1.533–2.208)	<0.001

CI: confidence interval; HbA1c: hemoglobin A1c; NIHSS: National Institute of Health stroke scale

## Discussion

Based on our data, it is reasonable to speculate that an elevated HbA1c level on admission is adversely associated with functional outcome in patients with SAO. Statistical differences still existed after adjusting for additional risk factors. This result differs from the findings presented by Fang et al.[24], although it is partially consistent with findings from several other previous studies[9,10,25,26]. Fang et al. suggested that hyperglycemia was not associated with functional outcome in patients with lacunar stroke[24]. One explanation for this difference is that they measured fasting glucose levels, which may be affected by confounding variables[11]. In addition, the study by Fang et al. had a longer follow-up period than the current study. The longer follow-up showed more curative effects of glucose-lowering. Previous studies have suggested that poor pre-stroke glycemic control was associated with unfavorable outcome after acute ischemic stroke[25,26]. Our results confirm the findings of these studies; however, patients with ischemic stroke included in previous studies were not classified according to the TOAST stroke subtypes. Therefore, previous findings do not reflect the etiological characteristics of patients diagnosed with SAO because they may have included patients diagnosed with large-artery atherosclerosis or cardioembolic stroke.

The mechanism underlying the association between abnormal glucose metabolism and poor outcome is still unclear. Recently, the following hypotheses have become widely accepted: the pro-oxidative and proinflammatory state induced by hyperglycemia may cause direct neuronal toxicity[27]; the procoagulant state secondary to hyperglycemia may exacerbate poor blood supply to the brain[28]; persistent hyperglycemia can increase the infarction volume[29]; and, more importantly, diabetes and HbA1c level may be determinants of leukoaraiosis-predominant microangiopathy[19]and smaller lacunes(even infarct sizes<3 mm)[30]. These mechanisms may be interrelated, and thus exacerbate brain injury. Occlusions of small penetrating arteries in the deep brain and leukoaraiosis are mainly responsible for SAO stroke. Our findings can thus be interpreted by the mechanisms presented in the aforementioned studies.

Abnormal glucose metabolism is a significant factor for poor outcome following acute ischemic stroke. For patients with diabetes, the American Diabetes Association recommends HbA1c testing at least twice a year in patients who have stable glycemic control, and more frequently(quarterly assessment) in patients whose therapy has changed or who are not meeting glycemic goals[17,31].For stroke patients, regular monitoring and routine HbA1c testing is important in clinical practice, especially among SAO patients. This can enable identification of high-risk patients who may benefit from early intervention.

There are certain limitations to the current study, as it was retrospective and used data from a single hospital. Moreover, no death records were found during the follow-up period. Although SAO has the lowest mortality rate among stroke subtypes and the study had a short follow-up period, it is unlikely that there were no patient deaths during this time. In addition, patients with no data on HbA1c values at admission were not included in this study. Therefore, there may have been a selection bias, even though there was no significant association in the distribution of excluded patients and patients who were analyzed in the study. Regretfully, another limitation is related to the lack of data of HbA1c values during the follow-up period, although fasting glucose levels measured in the hospital were available. There was no statistically significant difference between patients with favorable and unfavorable outcomes with regard to fasting glucose values. Therefore, it seems reasonable to infer that our findings are reliable.

## Conclusions

In this study, elevated HbA1c level on admission was found to be adversely associated with functional outcome 3 months after stroke onset among patients presenting with SAO. Although further study is required, regular monitoring and routine HbA1c testing at admission is important and may benefit patients diagnosed with SAO.

## Supporting Information

S1 Table Minimal data set of the study. (XLS)
